# Epidemiology, Trends, Utilization Disparities, and Outcomes of Catheter Ablation and Its Association With Coronary Vasospasm Amongst Patients With Non-valvular Atrial Fibrillation: A Nationwide Burden of Last Decade

**DOI:** 10.7759/cureus.40649

**Published:** 2023-06-19

**Authors:** Siva Naga S Yarrarapu, Parth Shah, Beshoy Iskander, Andrea Mestre, Aditya Desai, Shiv Shah, Renu Bhandari, Abdul-Rahaman Adedolapo Ottun, Anmol Bharti, Deepika Vunnam, Abdelhakim Ouled Said, Ya-Ching Hsieh, Urvish K Patel, Vikramaditya Samala Venkata

**Affiliations:** 1 Internal Medicine, Monmouth Medical Center/Rutgers University, Long Branch, USA; 2 Hospital Medicine, Tower Health Medical Group, Reading, USA; 3 Internal Medicine, Bon Secours Mercy Health - St. Elizabeth Youngstown Hospital (NEOMED), Youngstown, USA; 4 Internal Medicine, Universidad del Rosario, Bogota, COL; 5 Internal Medicine, University of California Riverside School of Medicine, Riverside, USA; 6 Internal Medicine, Government Medical College, Surat, Surat, IND; 7 Medicine, Manipal College of Medical Sciences, Pokhara, NPL; 8 Internal Medicine, Presbyterian Hospital, Agogo, GHA; 9 Internal Medicine, University of Ghana, Accra, GHA; 10 Internal Medicine, University College of Medical Sciences, Delhi, IND; 11 Internal Medicine, Dr. Pinnamaneni Siddhartha Institute of Medical Sciences and Research Foundation, Chinna Avutapalli, IND; 12 Internal Medicine, Hospital Ibn Sina, Rabat, MAR; 13 Anesthesiology, Icahn School of Medicine at Mount Sinai, New York, USA; 14 Public Health and Neurology, Icahn School of Medicine at Mount Sinai, New York, USA; 15 Internal Medicine, Cheshire Medical Center/Dartmouth-Hitchcock Keene, Keene, USA

**Keywords:** outcomes, disparities, utilization, coronary vasospasm, atrial fibrillation, catheter ablation

## Abstract

Background: Catheter ablation (CA) is an important curative treatment for non-valvular atrial fibrillation (NVAF), however, nationwide data on its utilization and disparities is limited. Coronary vasospasm is a rare, life-threatening, peri-operative complication of CA with limited literature in Caucasians.

Methods: We performed a retrospective study on adult hospitalizations in the USA from 2007 to 2017 by obtaining the data from National Inpatient Sample. The primary endpoints of our study were to identify the utilization rate of CA, disparities in utilization, and study the outcomes associated with CA. The secondary endpoints of the study were to identify the incidence of coronary vasospasm amongst patients who underwent CA, evaluate their association, and identify the predictors of coronary vasospasm.

Results: From 35,906,946 patients with NVAF, 343641 (0.96%) underwent CA. Its utilization decreased from 1% in 2007 to 0.71% in 2017. Patients who underwent CA, compared to those without CA, fared better in terms of hospital length of stay, mortality rate, disability rate, and discharge to the non-home facility. Patients in the 50-75 years age group, Native Americans, those with private insurance, and median household income of 76-100th percentile were associated with higher odds of CA utilization. Urban teaching hospitals and large-bedded hospitals performed more ablations, while the Mid-West region fared lower than the South, the West, and the Northeast. The prevalence of coronary vasospasm was higher amongst CA in comparison without CA, however, in regression analysis, no significant association was demonstrated between CA and coronary vasospasm.

Conclusion: CA is an important treatment modality that is associated with improved clinical outcomes. Identification of factors associated with lower utilization of CA and its disparities will help to mitigate the burden associated with NVAF.

## Introduction

Atrial fibrillation (AF), the most common supraventricular tachyarrhythmia [1], poses a significant economic burden together with serious cardiovascular morbidity and mortality. It is associated with advanced age. With the average life expectancy of the global population increasing, its prevalence is only bound to escalate [2,3]. More than 12 million people in the US are estimated to have AF by the year 2030. Its age-adjusted mortality rate increased significantly from 18 to 22.3 per 100,000 population over the past decade, with more than 150,000 deaths per year and $6.65 billion spent on health care annually [2,4-7]. Diabetes, hypertension, smoking, obesity, and dyslipidemia are some of the other important risk factors for AF. Recently, coronary artery disease has been shown to play an important role in the pathogenesis and incidence of AF with new evidence demonstrating an interdependent relationship between the two [8-10].

Catheter ablation (CA), first described in 1981, has undergone several technological advancements over the past decades and has proven to be an important curative treatment for paroxysmal, persistent, and permanent AF. Its fundamental principle involves mapping the site of abnormal cardiac impulse generation and its focal ablation by using either cryotherapy or radiofrequency. Not only is it indicated for patients who develop intolerance or fail pharmacotherapy, it is now being considered as a first-line, early treatment strategy in a select group of asymptomatic patients [11-15]. Thus, determining the utilization rate over the past decade and the outcomes of CA was one of our study objectives.

Previous evidence has found racial differences in CA utilization, pointing to unnoticed factors that may be contributing to bias involving the management of AF patients [16]. Patient comorbidities, hospital characteristics and location, and socioeconomic disparities could be some of the factors involved. Additionally, large-scale studies are limited. Therefore, we aimed to evaluate the epidemiology, and utilization disparity of CA.

Although minimally invasive, this procedure is not without complications. The safety of CA hasn’t been well established with regard to the patient’s age and underlying comorbidities. Cardiac tamponade, atrioesophageal fistula formation, and pulmonary vein stenosis are some of the commonly reported complications [17-22]. Coronary vasospasm, although infrequent, is a life-threatening phenomenon that has been gaining more attention over the past few years [23]. It is a high-risk complication that can cause myocardial ischemia and can present in the form of angina, cardiogenic shock, ventricular fibrillation, and cardiac arrest. The published literature on its incidence, predictors, and outcomes in relation to CA is limited. Although observational studies were previously conducted in the Japanese population [24,25], racial differences between the Japanese and Caucasian populations in association with vasospastic angina make these findings difficult to interpret [26]. With an increasing proportion of AF patients undergoing early procedures, herein, we aim to identify the odds of vasospasm in association with the procedure in the US population and study its predictors and outcomes.

This study finds importance in helping us identify the factors associated with the utilization, healthcare disparities, and outcomes of CA, and secondarily, helps us better understand the complication of coronary vasospasm, which would promote better management of the patients undergoing CA.

## Materials and methods

Details of data

Data was obtained from the Agency for Healthcare Research and Quality's Healthcare Cost and Utilization Project (HCUP) Nationwide Inpatient Sample (NIS) files between January 2007 and December 2017. The NIS is the largest publicly available all-payer inpatient care database in the United States and contains discharge-level data provided by states that participate in the HCUP (including a total of 46 in 2011). This administrative dataset contains data on approximately eight million hospitalizations in 1,000 hospitals that were chosen to approximate a 20% stratified sample of all US community hospitals, representing more than 95% of the national population. Criteria used for stratified sampling of hospitals into the NIS include hospital ownership, patient volume, teaching status, urban or rural location, and geographic region. Discharge weights are provided for each patient discharge record, which allows extrapolation to obtain national estimates. Each hospitalization is treated as an individual entry in the database and is coded with one principal diagnosis, up to 24 secondary diagnoses, and 15 procedural diagnoses associated with that stay. Detailed information on NIS is available at http://www.hcup-us.ahrq.gov/db/nation/nis/nisdde.jsp.

Study population, type, and demographic characteristics of the population

We performed a retrospective observational study on adult hospitalizations in the USA from 2007 to 2017. Primary and secondary diagnoses of AF were identified using the International Classification of Diseases (ICD-9) and 10-CM codes. AF was defined by 427.31, I48.0X (paroxysmal AF), I48.1X (persistent AF), and I48.2X (chronic AF). We have obtained patient characteristics of interest (age, sex, race, insurance status, admission day, admission type, median household income category, and concomitant diagnoses), hospital characteristics (hospital size, hospital region, and teaching versus nonteaching hospital), and concurrent conditions/comorbidities. Hospital region is based on the classification system given by the US Census Bureau on "region" which divides the US into West, Midwest, South, and Northeast. Hospital size is based on the number of beds and divided the hospitals into small (fewer than 100 beds), medium (100 to 499 beds), and large (500 or more beds).

We identify comorbidities of hypertension, diabetes mellitus, obesity, dyslipidemia, abuse/dependence on drugs, alcohol and tobacco, renal failure, HIV/AIDS, solid tumor, depression, ischemic heart disease, AF, and congestive heart failure (CHF) using ICD 9 and 10 codes. Age <18 years and admissions with missing data for age, sex, and race were excluded.

The comparison was made between AF undergoing CA and without CA. CA was defined by ICD-9 and 10 procedure codes as a primary procedure (ICD-9-CM Procedure 37.34 and ICD-10 procedure codes 02583ZZ, 02563ZZ, 02573ZZ, 025K3ZZ, 025L3ZZ, 02B63ZZ, 02B73ZZ, 02BK3ZZ, 02BL3ZZ).

Endpoints

The primary endpoints of our study were to identify the utilization rate of CA, disparities in utilization, and study the outcomes associated with CA. The secondary endpoints of the study were to identify the incidence of coronary vasospasm amongst patients who underwent CA, evaluate their association, and identify the predictors of coronary vasospasm.

Coronary vasospasm was defined as ICD-9 and 10 (413.1 and I20.1) as new events during hospitalization. We have included complications like post-procedural bleeding (life-threatening and minor), cardiac complications, respiratory, and infections. Outcomes were defined as all-cause in-hospital mortality, hospital stay, cost, discharge disposition, and disability. Discharge disposition was divided into two groups (home vs non-home discharge) and disability was calculated based on All Patient Refined DRGs (APR-DRGs)_severity of disease, developed by 3M Health Information Systems (0-4: No, Minor, Moderate, Major, and Extreme loss of function).

Statistical analysis

All statistical analyses were performed using the weighted survey methods in Statistical Analysis Software (SAS) (version 9.4, SAS Institute, North Carolina State University, USA). Univariate and bivariate analysis of differences between categorical variables were tested using the chi-square test and analysis of differences between continuous variables were tested using unpaired student's t-test. The mixed-effects survey logistic regression models with weighted analysis were used for the dependent variables, to estimate the odds ratio (OR) and 95% confidence intervals to identify the association between coronary vasospasm and CA as well as predictors and outcomes of coronary vasospasm. p-values of <0.05 were considered significant and all statistical tests used were two-sided. No predetermined sample size was calculated or considered. For each model, the C-index (a measure of goodness of fit for the logistic regression model) was calculated.

Details of Confounders/Variables

We have adjusted regression analysis with demographics of patients (age, sex, race), patient characteristics (admission day, primary payer, admission type, and median household income category), comorbidities (hypertension, diabetes mellitus, obesity, dyslipidemia, abuse/dependence of drug, alcohol and tobacco, renal failure, HIV/AIDS, solid tumor, depression, ischemic heart disease, AF, and CHF), and hospital characteristics (hospital region, teaching versus nonteaching hospital, hospital bed size).

## Results

Disease hospitalizations

The total unweighted hospitalizations for AF was 10,565,495 hospitalizations and the total weighted was 52,278,160 hospitalizations from 2007 to 2017. After excluding patients <18 years of age and patients with missing demographic data such as age, gender, and race, the total number of AF patients was 35,906,946 hospitalizations. CA procedure was performed in 343,641 (0.96%) patients.

Prevalence trends

**Figure 1:**
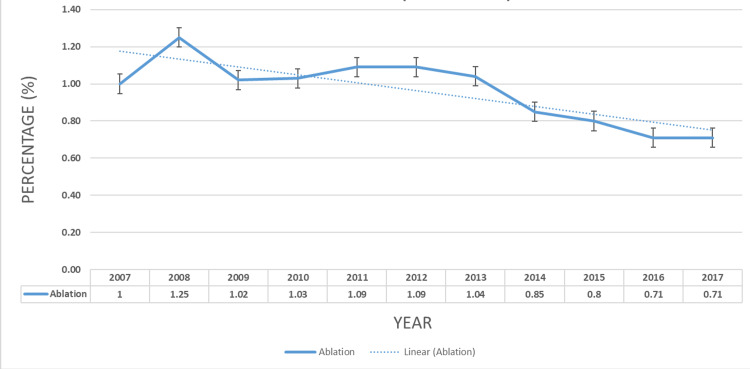
Prevalence trend of catheter ablation in US hospitalizations from 2002 to 2017 (Ptrend <0.0001)

The trend for CA for AF hospitalizations was analyzed. This trend is shown in Figure 1 where it was decreasing from 1% in 2007 to 0.71% in 2017, Ptrend<0.0001.

Demographics, patient comorbidities, and hospital characteristics

Of 35,906,946 patients with non-valvular atrial fibrillation (NVAF), 343641 (0.96%) underwent CA. Patients who underwent CA were mostly in the age group of 50-75 years (63.67%) vs <50 years (8.63%) vs >75 years (27.71%). The majority of patients undergoing CA were men (61.6%) and was more common amongst White race (86.17%) vs Native American (0.58%) vs Black (7.31%). Patients with a median household income falling in the 76th-100th percentile (28%) were the majority to have the procedure. Most of the patients had Medicare insurance (59.5%) vs Medicaid (4.06%) vs private insurance (32.71%). CA was performed more often during weekdays (89.54%) vs weekends (10.46%) and frequently done as a non-elective procedure (51.41%) vs elective (48.59%). It was more frequent in urban teaching hospitals (73.14%) vs urban nonteaching (24.1%) and was more common in the South region (39.57%) vs the West region (19.33%). Co-morbidity comparison between the cohorts with vs without CA include hypertension (67.4% vs 75.52%), diabetes (24.53% vs 33.8%), CHF (33.4% vs 42.45%), alcohol use (2.12% vs 3.17%), tobacco use (7.38% vs 7.72%), and drug abuse (1.02% vs 1.37%). p-value was noted to be <0.0001 for the above comparisons (Table 1).

**Table 1:** Characteristics of patients undergoing catheter ablation amongst US hospitalizations (2007-2017) TIA: transient ischaemic attack; AIS: acute ischemic stroke

Variables	Catheter Ablation N=343,641 (0.96%)	No-Catheter Ablations N=35,563,304 (99.04%)	Total N= 35,906,946 (100%)	p-value
Demographic and Socioeconomic Characteristics (%)				
Age Groups				
Age Group 18-50 years	29,639 (8.63)	1,098,655 (3.09)	1,128,295 (3.14)	<0.0001
Age Group 50-75 years	218,781 (63.67)	13,380,000 (37.63)	13,600,000 (37.87)	<0.0001
Age Group >75 years	95,219 (27.71)	21,080,000 (59.29)	21,180,000 (58.98)	<0.0001
Sex				
Male	211,667 (61.6)	17,890,000 (50.3)	18,100,000 (50.41)	<0.0001
Female	131,970 (38.4)	17,670,000 (49.7)	17,810,000 (49.59)	<0.0001
Race				
White	289,183 (86.17)	29,270,000 (84.03)	29,560,000 (84.05)	<0.0001
Black	24,535 (7.31)	2,941,361 (8.44)	2,965,897 (8.43)	<0.0001
Hispanic	15,417 (4.59)	1,833,665 (5.26)	1,849,082 (5.26)	<0.0001
Asian or Pacific Islander	4,533 (1.35)	643,373 (1.85)	647,907 (1.84)	<0.0001
Native American	1,937 (0.58)	143,517 (0.41)	145,454 (0.41)	<0.0001
Median household income for patient's ZIP Code				
0-25th percentile	74,740 (22.2)	9,124,548 (26.13)	9,199,289 (26.09)	<0.0001
26th to 50th percentile (median)	81,482 (24.2)	9,046,942 (25.91)	9,128,425 (25.89)	<0.0001
51st to 75th percentile	86,202 (25.6)	8,589,608 (24.6)	8,675,810 (24.61)	<0.0001
76th to 100th percentile	94,258 (28)	8,157,289 (23.36)	8,251,548 (23.41)	<0.0001
Patient level characteristics (%)				
Primary Payer (%)				
Medicare	204,261 (59.5)	28,570,000 (80.44)	28,770,000 (80.23)	<0.0001
Medicaid	13,952 (4.06)	1,396,179 (3.93)	1,410,132 (3.93)	<0.0001
Private Insurance	112,290 (32.71)	4,483,255 (12.62)	4,595,546 (12.81)	<0.0001
Other/Self-pay/No charge	12,774 (3.72)	1,069,819 (3.01)	1,082,593 (3.02)	<0.0001
Admission type (%)				
Non-elective	175,944 (51.41)	30,250,000 (85.3)	30,430,000 (84.97)	<0.0001
Elective	166,300 (48.59)	5,214,433 (14.7)	5,380,732 (15.03)	<0.0001
Admission day (%)				
Weekday	307,698 (89.54)	27,900,000 (78.46)	28,210,000 (78.57)	<0.0001
Weekend	35,942 (10.46)	7,659,189 (21.54)	7,695,132 (21.43)	<0.0001
Concurrent comorbidities (%)				
Hypertension (%)	231,625 (67.4)	26,860,000 (75.52)	27,090,000 (75.44)	<0.0001
Diabetes mellitus (%)	84,307 (24.53)	12,020,000 (33.8)	12,110,000 (33.71)	<0.0001
Metabolic Syndrome (%)	686 (0.2)	68,822 (0.19)	69,508 (0.19)	<0.4131
Dyslipidemia (%)	930 (0.27)	84,864 (0.24)	85,794 (0.24)	<0.0001
Abuse/dependence of drug (%)	3,490 (1.02)	485,725 (1.37)	489,215 (1.36)	<0.0001
Abuse/dependence of alcohol (%)	7,294 (2.12)	1,126,088 (3.17)	1,133,383 (3.16)	<0.0001
Abuse/dependence of tobacco (smoking) (%)	25,349 (7.38)	2,745,887 (7.72)	2,771,236 (7.72)	<0.0001
Congestive heart failure (%)	114,840 (33.42)	15,100,000 (42.45)	15,210,000 (42.36)	<0.0001
TIA (%)	888 (0.26)	382,950 (1.08)	383,839 (1.07)	<0.0001
AIS (%)	517 (0.15)	1,021,605 (2.87)	1,022,122 (2.85)	<0.0001
Hospital level characteristics (%)				
Bed Size of hospital (%)*				
Small	24,319 (7.13)	5,502,324 (15.52)	5,526,643 (15.44)	<0.0001
Medium	68,905 (20.2)	9,603,313 (27.09)	9,672,218 (27.03)	<0.0001
Large	247,914 (72.67)	20,340,000 (57.38)	20,590,000 (57.53)	<0.0001
Hospital Location & Teaching Status (%)				
Rural	9,417 (2.76)	3,999,458 (11.28)	4,008,876 (11.2)	<0.0001
Urban Non-teaching	82,199 (24.1)	13,580,000 (38.31)	13,660,000 (38.17)	<0.0001
Urban Teaching	249,522 (73.14)	17,870,000 (50.41)	18,120,000 (50.62)	<0.0001
Hospital Region (%)				
Northeast	85,247 (24.81)	8,188,389 (23.02)	8,273,636 (23.04)	<0.0001
Midwest	55,982 (16.29)	7,346,860 (20.66)	7,402,843 (20.62)	<0.0001
South	135,973 (39.57)	13,690,000 (38.49)	13,820,000 (38.5)	<0.0001
West	66,438 (19.33)	6,340,184 (17.83)	6,406,623 (17.84)	<0.0001

Outcomes of CA in AF hospitalizations

Table 2 shows the outcomes of AF patients who had undergone CA. Outcomes were discharge, mortality, disability/loss of function, cost, and length of stay (LOS).

**Table 2:** Univariate analysis of the outcomes in catheter ablation and no-catheter ablation @Disability: APR-DRG severity was defined by APR-DRG: All Patients Refined-Diagnosis Related Groups

Outcomes	Catheter Ablation N=343,641 (0.96%)	No-Catheter Ablations N=35,563,304 (99.04%)	Total N= 35,906,946 (100%)	p-value
Discharge to non-home (%)	17.99	54.01	53.65	<0.0001
In hospital all cause mortality (%)	0.76	5	4.96	<0.0001
Severe and extreme disability (%) @	32.49	57.04	56.8	<0.0001
Length of stay (days)	4.6	6.0		<0.0001
Cost of hospitalization ($)	111,711	53,930		<0.0001

Patients who underwent CA compared to those without CA fared better in terms of mean length of hospital stay (4.64 days vs 6.02 days, p<.0001), the mortality rate (0.76% vs 5%, p<0.0001), disability rate (32.49% vs 57.04%, p<0.0001), discharged to the non-home facility (17.99% vs 54.01%, p<0.0001), however, mean cost of hospitalization was higher in CA patients ($111,711 vs $53,930, p<.0001).

Outcomes of CA in AF hospitalizations - multivariable regression analysis model

Table 3 lists the multivariable regression analysis models for death, discharge, disability, morbidity, and risk of death. After adjusting for various variables such as age, sex, race, etc., the models showed that patients who had undergone CA had lower odds of death (adjusted OR 0.196, 95% CI 0.179-0.214, p<.0001), discharge to non-home (adjusted OR 0.268, 95% CI 0.263-0.274, p<.0001), severe and extreme disability (adjusted OR 0.418, 95% CI 0.411-0.425, p<.0001), morbidity (adjusted OR 0.473, 95% CI 0.456-0.492, p<.0001), and risk of death (adjusted OR 0.578, 95% CI 0.568-0.588, p<.0001), with AUC or C statistic of 0.633, 0.69, 0.682, 0.615, and 0.676, respectively.

**Table 3:** Outcomes of catheter ablation in atrial fibrillation hospitalizations - multivariable regression analysis model * Morbidity is defined by - hospital stay >90th Percentile of mean hospitalization + discharge to non-home (home health care, skilled nursing facility, assisted living facility) @Disability: APR-DRG severity was defined by # Risk of death: APR-DRG risk of mortality was defined by APR-DRG: All Patients Refined-Diagnosis Related Groups

Effect	Odds Ratio (OR)	95% Confidence Limits		P Value	Area under the ROC Curve/c-index
		Lower Limit	Upper Limit		
Model 1: Death	0.196	0.179	0.214	< .0001	0.633
Model 2: Morbidity*	0.473	0.456	0.492	< .0001	0.615
Model 3: Discharge to non-Home	0.268	0.263	0.274	< .0001	0.690
Model 4: Disability @	0.418	0.411	0.425	< .0001	0.682
Model 5: Risk of death #	0.578	0.568	0.588	< .0001	0.676

Utilization of CA and its disparities

Table 4 shows the multivariable analysis of predictors of utilization among inpatients with AF.

**Table 4:** Regression analysis showing predictors of utilization of catheter ablation TIA: transient ischaemic attack; AIS: acute ischemic stroke; CHF: congestive heart failure; HTN: hypertension; TG: triglyceride

	Odds Ratio (OR)	95% Confidence Limits		P value
		Lower Limit	Upper Limit	
Age				
18-50 years	Reference			
50-75 years	1.592	1.525	1.662	<0.0001
>75 years	1.159	1.089	1.662	<0.0001
Race				
					White	Reference				
African American	0.695	0.674	0.718	<0.0001	
Hispanic	0.788	0.759	0.819	<0.0001	
Asian or Pacific Islander	0.609	0.568	0.652	<0.0001	
Native American	1.39	1.245	1.552	<0.0001	
Sex					
Female	Reference				
Male	0.883	0.869	0.898	<0.0001	
Median Household income by Zipcode					
0-25th percentile	Reference				
26th-50th percentile	1.073	1.048	1.098	<0.0001	
51-75th percentile	1.077	1.052	1.103	<0.0001	
76-100th percentile	1.154	1.126	1.182	<0.0001	
Primary Payer					
Medicare	Reference				
Medicaid	0.687	0.658	0.718	<0.0001	
Private insurance	1.307	1.282	1.333	<0.0001	
Other/self-pay/no charge	0.854	0.817	0.892	<0.0001	
Admission Type					
Elective	Reference				
Non-elective	3.958	3.894	4.024	<0.0001	
Admission Day					
Weekday	Reference				
Weekend	0.683	0.665	0.701	<0.0001	
Bed Size of Hospital					
Small	Reference				
Medium	1.632	1.578	1.688	<0.0001	
Large	2.681	2.6	2.763	<0.0001	
Hospital Location and Teaching Status					
Rural	Reference				
Urban non-teaching	2.451	2.336	2.571	<0.0001	
Urban teaching	5.092	4.861	5.334	<0.0001	
Hospital Region					
Northeast	Reference				
Midwest	0.717	0.698	0.735	<0.0001	
South	1.01	0.989	1.032	0.3428	
West	1.059	1.034	1.085	<0.0001	
Comorbidities of Patients					
Smoking	0.723	0.701	0.745	<0.0001	
Drug	0.571	0.527	0.618	<0.0001	
Alcohol	0.514	0.486	0.542	<0.0001	
TIA	0.392	0.336	0.456	<0.0001	
AIS	0.087	0.071	0.106	<0.0001	
CHF	1.069	1.051	1.087	<0.0001	
HTN	0.869	0.854	0.884	<0.0001	
Diabetes	0.661	0.648	0.673	<0.0001	
HIGH TG	0.905	0.78	1.051	0.1905	
Area under the ROC curve/c-index	0.804				

Patients in 50-75 years age group (OR 1.59, 95% CI 1.52-1.66, p<0.0001), Native Americans (1.39, 1.24-1.55, p<0.0001), those with private insurance (1.30, 1.28-1.33, P<0.0001), and with a median household income of 76-100th percentile (1.15, 1.12-1.18, P<0.0001) were associated with higher odds of CA utilization. Its use was lower in males (0.88, 0.86-0.89, p<0.0001) than in females. CA was performed more on weekdays compared to weekends (0.68, 0.66-0.70, P<0.0001) and as a nonelective procedure (3.95, 3.89-4.02, P<0.0001). While urban teaching hospitals (5.09, CI: 4.86-5.33, P<0.0001) and large-bedded hospitals (2.68, 2.60-2.76, P<0.001) performed more ablations, the Mid-West region (OR 0.71, 95% CI: 0.69-0.73, P<0.001) fared lower than the South, the West, and the North-East. Patients with CHF had seen higher odds of utilization (1.06, 1.05-1.08, P<0.0001), whereas HTN (0.86, 0.85-0.88, P<0.0001), smoking (0.72, 0.70-0.74, P<0.0001), diabetes mellitus (0.66, 0.64-0.67, P<0.0001), drug usage (0.57, 0.52-0.61, P<0.0001), alcohol consumption (0.51, 0.48-0.54, P<0.0001), TIA (0.39, 0.336-0.45, P<0.0001), and AIS (0.08, 0.07-0.10, P<0.0001) were associated with lower odds.

The AUC or C statistic was 0.804.

Predictors of vasospasm in AF hospitalizations

Table 5 lists a multivariable analysis of the predictors of vasospasm in AF hospitalizations.

**Table 5:** Regression analysis showing predictors of coronary spasm following catheter ablation TIA: transient ischaemic attack; AIS: acute ischemic stroke; CHF: congestive heart failure; HTN: hypertension; TG: triglyceride

Odds Ratio Estimates				
	Odds Ratio (aOR)	95% Confidence Limits		P Value
		Lower Limit	Upper Limit	
Procedure				
No catheter ablation	Reference			
Catheter ablation	1.294	0.826	2.027	0.2599
AGE (every 10 years)	0.957	0.953	0.961	<0.0001
Race				
White	Reference			
African American	1.008	0.831	1.222	0.9377
Hispanic	0.982	0.758	1.272	0.8916
Asian or Pacific Islander	1.217	0.825	1.796	0.3212
Native American	0.909	0.371	2.224	0.8342
Sex				
Female	Reference			
Male	2.043	1.807	2.309	<0.0001
Median Household Income By Zip Code				
0-25th percentile	Reference			
26th-50th percentile	1.249	1.057	1.476	0.0089
51-75th percentile	1.225	1.027	1.46	0.0242
76-100th percentile	1.229	1.021	1.479	0.0294
Primary Payer				
Medicare	Reference			
Medicaid	1.02	0.805	1.293	0.8687
Private insurance	1.107	0.936	1.309	0.2345
Other/self-pay/no charge	0.912	0.676	1.23	0.5448
Admission Type				
Elective	Reference			
Non-elective	0.835	0.702	0.994	0.042
Admission Day				
Weekday	Reference			
Weekend	1.152	1.003	1.323	0.0447
Bed Size of Hospital				
Small	Reference			
Medium	1.239	1.014	1.516	0.0366
Large	1.278	1.063	1.538	0.0091
Hospital Location and Teaching Status				
Rural	Reference			
Urban non-teaching	0.876	0.698	1.098	0.2503
Urban teaching	1.115	0.9	1.381	0.3179
Hospital Region				
Northeast	Reference			
MIdwest	1.242	1.031	1.497	0.0228
South	1.082	0.911	1.284	0.3686
West	1.309	1.08	1.587	0.0061
Comorbidities of Patients				
smoking	1.034	0.861	1.242	0.7198
Drug	3.363	2.685	4.212	<0.0001
Alcohol	0.884	0.672	1.164	0.3797
TIA	1.113	0.642	1.929	0.704
AIS	0.31	0.165	0.58	0.0002
CHF	0.565	0.493	0.648	<0.0001
HTN	1.254	1.088	1.446	0.0018
Diabetes	0.678	0.59	0.779	<0.0001
HighTG	1.868	0.885	3.942	0.1011
Area under the ROC curve/c-index			0.504	

The prevalence of coronary vasospasm was higher amongst CA in comparison to those without CA (0.03% vs 0.02%; p<0.0001). In regression analysis, we found a non-significant association between CA and coronary vasospasm (aOR 1.29, 95% CI 0.83-2.03; p=0.2599). Predictors associated with coronary vasospasm were male (2.04, 1.80-2.31, p<0.0001), non-elective procedure (0.84, 0.70-0.99, p=0.042), weekend procedure (1.15, 1.003-1.323, p=0.0447), substance abuse (3.36, 2.68-4.21, p<0.0001), and hypertension (1.25, 1.09-1.45, p-0.0018). Other comorbidities that appeared to be protective include diabetes (0.68, 0.59-0.78, p<0.0001), CHF (0.565, 0.49-0.65, p<0.0001), and acute ischemic stroke (0.31, 0.17-0.58, p=0.0002).

The AUC or C statistic was used to validate the accuracy of the regressions, which in this model is 0.504.

## Discussion

CA is an effective management strategy for AF patients who fail/develop intolerance to medical treatment, with the European Society of Cardiology (ESC) guidelines 2020 denoting it as a second-line therapy [27]. But in 2012, the first randomized clinical trial to document the superiority of early treatment with radiofrequency CA over anti-arrhythmic drugs was executed in 294 patients with paroxysmal AF [28]. A significant decrease in AF burden was documented at 24 months post-procedure in the ablation group. The findings were even reciprocated with cryoablation for the initial management of paroxysmal AF. This was documented in a randomized controlled trial of 303 patients where the ablation group had a lower incidence of recurrent/persistent atrial arrhythmias compared to the antiarrhythmic group over a three-year follow-up [29]. The advantage of early cryoablation over medical therapy was likewise demonstrated by Wazni et al., with a lower recurrence of atrial arrhythmias [30]. The recently published “EAST AFNET4” (Early Treatment of Atrial Fibrillation for Stroke Prevention Trial) trial discussed the potential advantages of initiating the early rhythm-control strategy (which included management with CA) in patients with AF when compared to standard care [31]. It demonstrated a lower frequency of adverse cardiovascular events in the rhythm-control group. Another recently discovered advantage of CA might be a lower incidence of dementia as reported in certain studies [32,33]. Thus, there is scope for CA to be considered as first-line therapy for AF in future guidelines if the results can be matched in large studies with good generalizability.

In our study, the outcomes of CA such as odds of death, discharge to non-home, severe disability, morbidity, and risk of death were found to have statistically improved outcomes compared to no ablation group. This explains the decrease in the burden of AF after CA [34].

Demographic data of our study suggest that the majority of the population undergoing CA were younger (50-75 years), males, and belonging to the Native American race. Recent studies have shown that Native Americans may have the highest incidence of AF. Of late, a large longitudinal analysis conducted in California, demonstrated this association when the annual incidence of AF was the highest in Native Americans, among all the races, despite controlling for sociodemographic and co-morbidities [35]. A previous study also demonstrated similar findings, where the Native American race had the highest prevalence of AF [36]. It is yet to be determined if there is a genetic predisposition or acquired exposure to risk factors that might have increased their incidence.

As with any planned procedure, the majority of CA was performed on weekdays and as a non-elective procedure. Socioeconomic status was a key factor in determining the likelihood of a patient undergoing CA. Patients with a median household income of 76-100th percentile, and private insurance had the highest odds of utilization. This was likewise demonstrated in previous studies that reported higher socioeconomic status as a key factor for undergoing the procedure [37].

The overall trend for CA utilization in US hospitalizations was found to be decreasing from 2007 to 2017. Rojulpote et al. studied the trends of CA utilization in the US between 2004 and 2014 among different income classes. It was found that the trend has been decreasing among patients with a low-income status but increasing in patients with higher incomes [38].

While urban teaching hospitals and large bedded hospitals performed more ablations, the Southern region had the most ablations compared to the West, Mid-West, and North-East. The Mid-West had the lowest odds of utilization compared to the remainder of the US. The Southern region was shown to have the greatest prevalence of cardiovascular risk factors as well as cardiovascular mortality in prior studies, which could potentially reflect the number of ablations in the South in our study. South-eastern US was also found to have the maximum healthcare utilization. Sinner et al. investigated the utilization of CA between 2007 and 2009 and correlated the regional prevalence of AF to healthcare spending. He found varied regions distributed throughout the US with the highest utilization of CA [39,40].

Patients with CHF had seen higher odds of utilization. About 42% of the patients with AF have coexistent CHF [41]. CA in this population was demonstrated previously to markedly improve mortality compared to medical therapy in a multicenter randomized controlled trial [42]. The observed benefit in improving mortality was also noticed in patients with heart failure with reduced ejection fraction (HFrEF) [43]. Whereas hypertension, smoking, diabetes mellitus, drug usage, alcohol consumption, TIA, and AIS were associated with lower odds of utilization. This could potentially be related to a lower success rate or a high risk of atrial arrhythmia recurrence in patients with severe comorbidities. D'Angelo et al. described how healthier patients had a higher likelihood of undergoing the procedure on account of similar reasons [37].

Coronary vasospasm is a rare but life-threatening complication of CA that can sometimes culminate in sudden cardiac arrest (SCA) [24,44,45]. Its proposed pathophysiology of CA is multifactorial yet not well-defined. It includes transmural inflammation of the myocardium or changes in the autonomic activity of ganglionated plexus in epicardial adipose tissue via thermal injury, or vasospasm induced by medications [40]. By far, there have only been a handful of documented cases and a couple of observational studies about coronary vasospasm materializing as a rare peri-operative complication of the procedure [24-26]. The literature also highlights late-onset coronary vasospasm documented in association with the procedure [45]. However, it is unknown whether the procedure increases the incidence of vasospasm as a long-term effect in the presence of other risk factors. Also, none of the above-mentioned observational studies were conducted in the Caucasian population. We used real-world data in our study to analyze the association between in-hospital AF treated with CA and coronary artery vasospasm. Our study is the first of its kind in this regard that tried to investigate this association by working on a retrospective cohort analysis. The odds of coronary vasospasm were found to be 29% higher in the CA cohort versus no ablation, albeit, having a statistically non-significant association. The underlying reason could be related to the low prevalence of coronary vasospasm cases in the CA cohort, thus having very limited power in demonstrating the association or detecting the difference between the two cohorts. A Japanese Cohort analyzed periprocedural coronary vasospasm in patients undergoing CA for AF, however, they were not able to demonstrate an association [26]. Another multicenter trial by Nakamura et al. was also unable to comment on the association [25]. These two studies involved 22,232 and 2,913 patients respectively that found coronary vasospasm to have <1% (0.19% and 0.31% respectively) incidence of CA; however, it was carried out in the Japanese population [25,26]. Given the significance of racial disparity in vasomotor reactivity of coronary arteries, our study helps provide a better comprehension of the relation between vasospasm and CA in the US population.

Substance abuse was found to elevate the risk of vasospasm by more than 300% in our study. This is in concordance with the published studies that documented tobacco smoking, alcohol, amphetamines, and cocaine as causes of vasospasm [46]. Hypertension was noted to be a predictor (25% higher odds), quite the contrary to reported literature on this association. Chen et al. concluded a paradoxical relation between hypertension and vasospasm [47]. In a retrospective study on the Taiwanese population, hypertension was shown to have an inverse effect on vasospasm, especially in females with high C-reactive protein (CRP) levels [48]. Apart from them, diabetes and CHF appeared to be protective in our study. While diabetes mellitus is a major contributing factor to coronary atherosclerosis, it had previously been proven to not elevate the risk of vasospasm. This was established in a prospective study on diabetes patients with intracoronary acetylcholine provocation test in the Korean population [49]. Likewise, CHF has never been documented to have an associated risk with vasospasm. Additional investigations evaluating the link between hypertension and CHF on vasospasm in the CA sub-group, with long-term follow-up of patients, especially in the Caucasian population, are warranted.

Strengths and limitations

To the best of our knowledge, this is the largest retrospective study that looked at the epidemiology, utilization rates and disparities, and predictors of utilization of CA especially for NVAF. NIS is the largest inpatient database and so the study is highly representative of the USA. This study had a large sample size because of the large database and we can conclude that there is good statistical power. Ours is the first study to analyze the association between coronary vasospasm and CA in the US population. There was fair representation in terms of age group, race, sex, and socioeconomic status. The above characteristics could potentially make our study validated externally to the real-world population.

Like every study with strengths, there were some limitations in this study. Data from administrative databases was obtained via discharged codes, billing codes, etc., and hence they are susceptible to coding errors. Furthermore, because the study is retrospective, causality could not be established between vasospasm and the predictors. Finally, because this was an inpatient population-based study, there might be a risk of underreporting as not all NVAF patients are admitted and some are managed as out-patient cases.

## Conclusions

CA is an important treatment modality of NVAF that has been associated with improved outcomes in our study. Together with the recent investigations in the literature, our study reinforces the potential possibility of its utilization as a first-line therapeutic strategy for NVAF. Identification of factors associated with lower utilization of CA and its disparities will help to mitigate the burden associated with NVAF.
